# Evidence on the Preventive Effects of the Postbiotic Derived from Cow’s Milk Fermentation with *Lacticaseibacillus paracasei* CBA L74 against Pediatric Gastrointestinal Infections

**DOI:** 10.3390/microorganisms11010010

**Published:** 2022-12-20

**Authors:** Franca Oglio, Cristina Bruno, Serena Coppola, Roberta De Michele, Antonio Masino, Laura Carucci

**Affiliations:** 1 Department of Translational Medical Science, University of Naples Federico II, 80131 Naples, Italy; 2 ImmunoNutriton Laboratory at CEINGE Advanced Biotechnologies, University of Naples Federico II, 80131 Naples, Italy

**Keywords:** acute gastroenteritis, *Rotavirus*, SARS-CoV-2, COVID-19, gut microbiome, infants, butyrate, probiotics, gut barrier, fermented foods

## Abstract

Postbiotics are commonly defined as preparations of inanimate probiotics and/or their cellular components and/or their metabolites/end products that confer health benefits on the host. They have been suggested as a promising strategy to limit infectious diseases. Emerging evidence support the efficacy of the postbiotic derived from cow’s milk fermentation with the probiotic *Lacticaseibacillus paracasei* CBAL74 (FM-CBAL74) in preventing pediatric infectious diseases. We aimed at reviewing the evidence available.

## 1. Introduction

**Figure 1:**
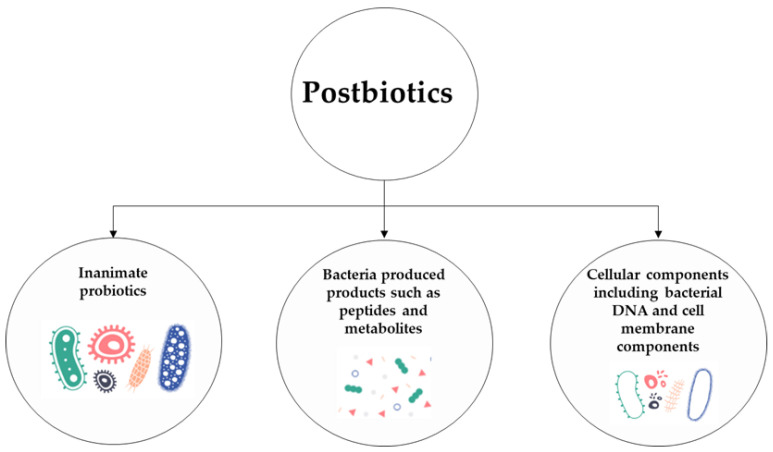
**Main active components of the postbiotics**. The positive effects elicited by the postbiotics on human health may result from the combined action of one or more components regulating different mechanisms. These effects could be related to the activities of the inanimate probiotics, bacterial components (i.e., lipoteichoic acid, peptidoglycans, bacteriocins, enzymes, nucleotides and/or peptides), and/or metabolites (such as short-chain fatty acids, SCFAs).

Postbiotics are defined as a “preparations of inanimate probiotics and/or their cellular components, and/or their metabolites/end products that confer health benefits to the host” [1]. Increasing evidence suggests the beneficial effects of postbiotics on human health highlighting the immunomodulatory, anti-inflammatory, anti-oxidant, and anti-viral properties [2] (Figure 1).

**Figure 2:**
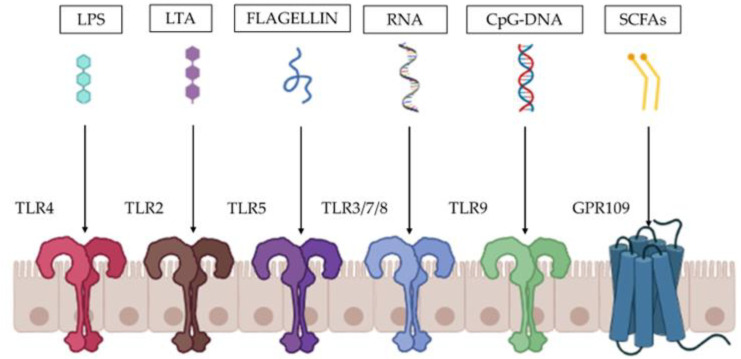
**Main receptors recognizing the postbiotic compounds at cell membrane level**. Different postbiotic compounds could bind different receptors located on the cell membrane of human cells.

These beneficial effects could be related to the activities of their components, including lipopolysaccharides (LPS), lipoteichoic acid (LTA), peptidoglycans, bacteriocins, enzymes, nucleotides, peptides, or short-chain fatty acids (SCFAs) [3]. Several components interact with the host’s immune system via Toll-like receptors (TRLs) as follows [4]: LPS are recognized by TLR4; LTA and peptidoglycans are recognized by TLR2; flagellin is recognized by TLR5; bacteria RNA can bind TLR3, TLR7, and TLR8, whereas bacterial CpG-DNA is recognized by TLR9 [5,6,7]. Another relevant postbiotics component is the SCFA butyrate that binds the G protein-coupled receptors such as GPR41, GPR43, GPR109, and other the nuclear receptors [8] (Figure 2).

As postbiotics do not contain living microorganisms, the risk of bacteremia or fungaemia associated with their use in human nutrition is minimized. Postbiotics lack the issue related to the development of antibiotic-resistance genes and the issue of living microorganism exposure to the immature immune system and gut barrier, especially in early life [9]. An assessment of safety for the intended use of any postbiotic is needed prior to use. Postbiotics derived from food-grade microorganisms in the EFSA QPS lists might be facilitated in the approval process.

From an economic point of view, an appealing aspect of postbiotics is related to high-temperature storage, facilitating shelf-life, packaging, and transportation [10]. In the Box 1, we summarized the strengths of the postbiotic approach.

Box 1 **Strengths of the postbiotic approach**

Health benefits similar to that obtained with living probioticsBetter safety profile than probioticsEfficient monitoring of technological process with high reproducibility of the biological effectsPossibility of being added to foods considered stressful to probiotics survival

## 2. The Postbiotic Derived from Cow Milk Fermentation with the Probiotic *L. paracasei* CBAL74

**Figure 3:**
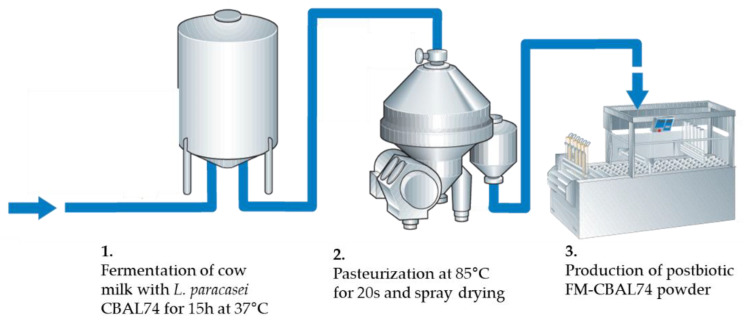
**Schematic representation of the multistep process for the synthesis of the postbiotic FM-CBAL74.** The FM-CBAL74 is obtained from skimmed cow milk fermentation with the probiotic *L. paracasei* CBAL74. The first step is a thermal processing, pasteurization of the skimmed cow milk to obtain enzymatic, and microbiological stability. Then the fermentation is started in the presence of 10^6^ CFU/gr *L. paracasei* CBAL74, reaching 5.9 × 10^11^ CFU/g after 15 h incubation at 37 °C. Then, the product is pasteurized at 85 °C for 20 s and spray dried. Thus, the final fermented milk powder contains only bacterial bodies and fermentation products, but not living microorganisms.

Fermented cow’s milk with *L. paracasei* CBAL74 (FM-CBAL74) is one of the most studied postbiotics in the pediatric age. This postbiotic derives from a well-defined cow milk fermentation process that is summarized in Figure 3. Briefly, skimmed cow’s milk powder is mixed with sterile water and then exposed to ultra-high temperature (UHT) treatment. Then the fermentation is started in the presence of 10^6^ CFU *L. paracasei* CBAL74 (International Depository Accession Number LMG P-24778), a probiotic included in the list of “Qualified Presumption of Safety microorganisms” drawn up by the Panel on Biological Hazards of the European Food Safety Authority, reaching 5.9 × 10^11^ CFU/g after 15 h incubation at 37 °C. The fermentation product is then pasteurized at 85 °C for 20 s for the inactivation of the living bacteria, and then spray dried. Thus, the final fermented milk powder contains only bacterial bodies and fermentation products, but not living microorganisms [11,12,13,14,15,16].

## 3. Preclinical Evidence on the Mechanisms of Action Elicited by FM-CBAL74

**Figure 4:**
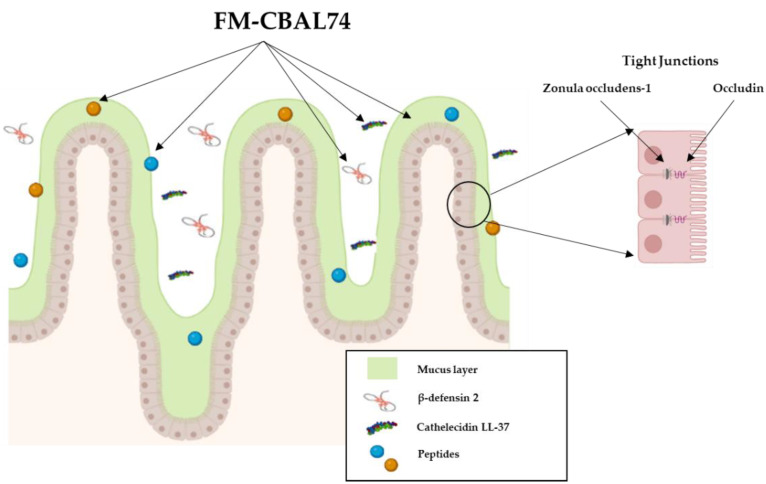
**FM-CBAL74 modulates different components of the epithelial gut barrier.** FM-CBAL74 promotes a beneficial modulation of epithelial gut barrier structure and function through a stimulation of enterocytes cell growth and differentiation, up-regulation of tight-junction proteins expression (i.e., zonula occludens-1 and occludin), mucus layer thickness (i.e., stimulation of mucin 2 expression), and innate immunity peptides release (i.e., β-defensin-2 and cathelicidin LL-37).

In vitro studies demonstrated that the postbiotic FM-CBAL74, through a direct interaction with human enterocytes, can exert beneficial actions on the major defense mechanisms against intestinal infections. Experiments using human enterocytes (Caco-2 cells) demonstrated that FM-CBAL74 was able to promote a beneficial modulation of cell growth and differentiation, tight-junctions proteins expression (i.e., zonula occludens-1 and occludin), mucus layer thickness (i.e., MUC5A expression), and innate immunity peptides production (i.e., β-defensin-2 and cathelicidin, LL-37) [11] (Figure 4).

It has been also shown an increase in Toll-like receptor 2 (TLR2), transcriptional factor nuclear factor kappa B1 (Nf- kB1), and trefoil factor 3 (TFF3) expression associated with a down-regulation of Toll-like receptor 4 (TLR4) expression after FM-CBAL74 exposure in human enterocytes, suggesting a potential anti-inflammatory activity of this postbiotic at intestinal level [11]. These effects were also confirmed in other experimental models, where FM-CBAL74 exerted a strong anti-inflammatory action on human dendritic cells in response to the *Salmonella typhimurium* infection. The effect derived mainly from an inhibition of pro-inflammatory cytokines release. Furthermore, it has been demonstrated that FM-CBAL74 has protective effects against DSS colitis in the animal model [17].

## 4. Preclinical Evidence on the FM-CBAL74 Effects against Intestinal Viral Infections

**Figure 5:**
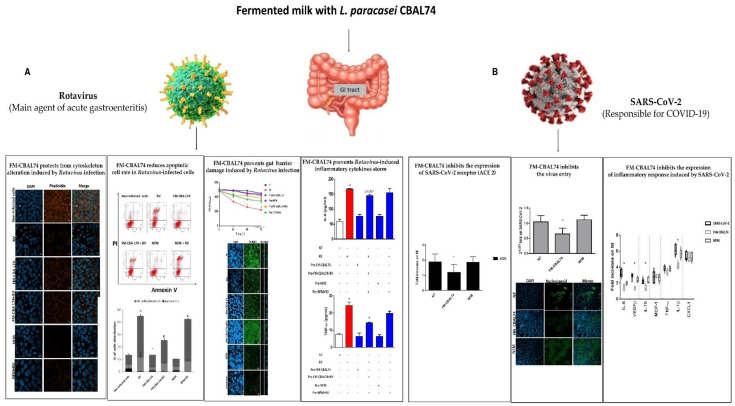
**Protective action of FM-CBAL74 against viral infections in human enterocytes**. (**Panel A**): FM-CBAL74 elicited beneficial effects on the gut barrier integrity preventing an RV-induced decrease in transepithelial electrical resistance and up-regulating the tight junction protein occludin, and reducing the RV-induced increase in IL-8 and TNF-α production in Caco-2 cells [12]. (**Panel B**): FM-CBAL74 was able to significantly reduce the number of infected enterocytes, as demonstrated by the reduction of nucleocapsid viral protein (N) positive cells, and to down-regulated ACE2 expression, preventing SARS-CoV-2 entry into human enterocytes. Furthermore, FM-CBAL74 was able to prevent the cytokine storm, inhibiting the IL-1β, IL6, vascular endothelial growth factor B, and IL-15 production induced by SARS-CoV-2 [13]. The symbol * means *p* < 0.05.

*Rotavirus* (RV) is the most common pathogen identified in children with acute gastroenteritis (AGE) worldwide [18]. It is a segmented double-stranded RNA virus with 11 genome segments encoding six structural proteins (VP) and six non-structural proteins (NSP). *Rotavirus* primarily infects mature enterocytes causing cell apoptosis and villus atrophy. Intestinal epithelial cells employ a range of defense strategies to counteract RV invasion, including mucus production and cytokines/chemokines expression [18]. The potential protective action elicited by FM-CBAL74 against RV infection has been recently investigated in a validated model of human enterocytes [12]. It has been demonstrated that FM-CBAL74 was able to protect against gut barrier damage, characterized by cytoskeleton alterations and apoptosis, involving the ERK/JNK kinase pathway. It has been reported that FM-CBAL74 had beneficial effects on the gut barrier integrity, preventing an RV-induced decrease in transepithelial electrical resistance and up-regulating the tight junction proteins, occludin, ZO-1, and the adherents’ junction protein, E-cadherin, and expression. Furthermore, FM-CBAL74 was able to prevent RV-induced oxidative stress (ROS production) and pro-inflammatory cytokines (IL-8, IL-6, and TNF-α) release, suggesting a potent inhibition of gut inflammation. These results suggest that the postbiotic FM-CBAL74 could be a disrupting nutritional strategy against RV-induced AGE (Figure 5A) [12]. The gastrointestinal tract is a target organ for SARS-CoV-2, the single-stranded RNA virus that is the agent of Coronavirus disease (COVID-19). Gastrointestinal symptoms (such as diarrhea, nausea, vomiting, anorexia, and abdominal pain) are commonly presented by COVID-19 pediatric patients [13]. It has been demonstrated that FM-CBAL74 was able to significantly reduce the number of infected enterocytes, as demonstrated by the reduction of Nucleocapsid (N) viral protein-positive cells, and to down-regulated ACE2 expression, preventing SARS-CoV-2 entry into human enterocytes. Furthermore, FM-CBAL74 was able to prevent the cytokine storm, inhibiting the IL-1β, IL6, vascular endothelial growth factor B (VEGF-B), and IL-15 production induced by SARS-CoV-2 (Figure 5B) [13].

## 5. Clinical Evidence on the Preventive Action of FM-CBAL74 against Pediatric Intestinal Infections

### 5.1. Clinical Data

The potential clinical efficacy of FM-CBAL74 in protecting children against acute gastroenteritis (AGE) has been explored in two multicenter randomized controlled trials (RCTs) registered in the Clinical Trials Protocol Registration System (ClinicalTrials.gov, accessed on 28 September 2022) [14,15]. We obtained the study protocol and the study reports containing all available raw data from the corresponding authors of both RCTs. The two RCTs showed a similar design. Briefly, healthy children (aged 12–48 months) attending daycare or preschool for at least five days a week during the winter season were randomized into the following two groups of 3-month dietary supplementation: FM-CBAL74 or placebo (maltodextrins). The study products were supplied in tins containing 400 g of powder, the packaging was similar, and storage was at room temperature in a dry environment. Family pediatricians instructed parents on the daily amount of the assigned product and how to prepare it. All subjects received 7 g/day of study products diluted in up to 150 mL of cow’s milk or water. The appearance and taste were the same for the two study products [16]. All children were formula fed and were invited to maintain the habitual diet, but to exclude prebiotics, probiotics, symbiotic, and immune-stimulating products during the 3-month study period.

**Figure 6:**
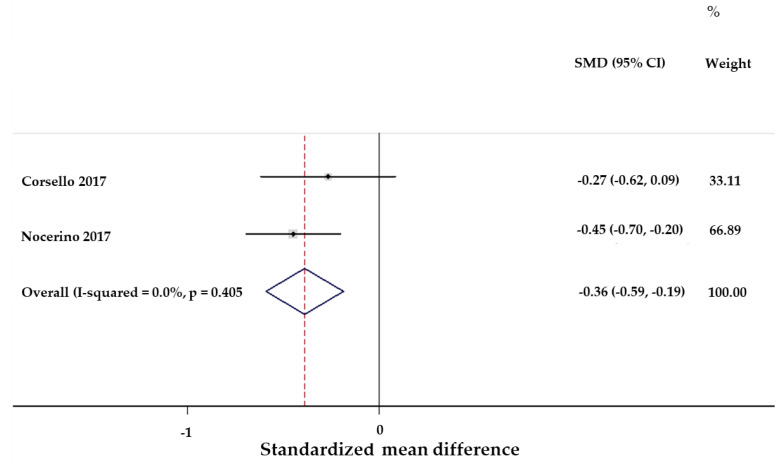
**Protective action by FM-CBAL74 against pediatric acute gastroenteritis: forest plot of the reduction of the absolute numbers of AGE episodes reported in two randomized clinical trials.** A meta-analysis of the raw data obtained by the corresponding author of the two randomized clinical trials was performed by applying the random-effects model. The heterogeneity between the two trials was assessed by Chi-squared and I-squared evaluation. The heterogeneity between the two studies was not significant with respect to the number of AGE episodes. Overall, when combining the outcome of both studies a significant impact by the dietary supplementation with the postbiotic on the total numbers of AGE throughout the trials was observed (Wald Chi-squared test). I-squared (variation in standardized mean difference attributable to heterogeneity) = 0.0%. *p* = 0.405. Test of SMD = 0: z = 3.77 *p* < 0.0001 [13,15].

Both trials revealed statistically significant differences in favor of the active group. In the first trial (evaluating 127 subjects in the FM-CBAL74 group and 141 subjects in the placebo group), the rate of children presenting at least one episode of AGE was lower in the FM-CBAL74 group (13.1% vs. 31.1%, *p* < 0.0001), and the total number of AGE episodes was lower in the FM-CBAL74 (21% vs. 47%, *p* < 0.0001) compared to the placebo group [14]. Similar results were reported by the authors of the second trial (involving 66 subjects in the active group and 60 in the placebo group), where the rate of children presenting at least one episode of AGE was lower in the FM-CBAL74 group (18.2% vs. 40.0%, *p* < 0.007), and the total number of AGE episodes was also lower in the active group compared with the placebo group (19% vs. 28%, *p* < 0.007) (Figure 6) [15].

### 5.2. Safety

Preclinical and clinical studies showed that the FM-CBAL74 formula, was safe and well-tolerated, and no adverse events were reported during the two clinical trials [14,15,16,17].

### 5.3. Fecal Immune Biomarkers and Microbiome Data

Study groups were also compared for fecal levels of α- and β-defensins, LL-37, and secretory immunoglobulin A (sIgA) at enrollment and after 3 months of intervention. In both trials, the authors observed an immunostimulatory effect consisting of a significant increase in innate (1–3: α- and β-defensins and LL-37) and adaptive immunity (sIgA) peptides production at the intestinal level [14,15].

Interestingly, analyzing the raw data of these two trials through a Spearman’s correlation rank test, we found a significant inverse correlation between an alpha-defensin increase in the GI tract and decreased risk to develop AGE. 

In a randomized-controlled trial, it has been also demonstrated that FM-CBAL74 dietary supplementation could exert a beneficial modulation of gut microbiome structure and function in formula-fed neonates/infants [16]. Newborns were randomly allocated to receive for the first trimester of life standard formula alone or standard formula supplemented with FM-CBAL74 (2.3 g/100 g, equivalent to 0.3% in ready-to-use infant formula). The reference group consisted only of breastfed infants. The results of this trial showed that FM-CBAL74 elicited a significant stimulation of the intestinal production of sIgA associated with a beneficial modulation of gut microbiome structure (i.e., increased abundance of SCFAs- producing bacteria) and function (i.e., decreased production of aspartic acid, methionine, arabitol, mannobiose, 2-3-dihydroxy-2-methyl-propiannoic acid, propylene glycol, pentanediol acid, stearic acid, galactofuranose and ribose, and increased production of sorbose, rhamnose, and fatty acids such as dodecanoic acid) [16].

Another study investigated the potential impact of FM-CBAL74 dietary supplementation on gut microbiome structure and function in healthy children (aged 1–4 years) [18]. After 3-month FM-CBAL74 dietary supplementation, the relative proportion of *Lactobacillaceae* and *Ruminococcaceae* increased significantly, with a significant specific increase in *Oscillospira* and *Faecalibacterium*. A positive correlation between the relative presence of several genera belonging to the *Ruminococcaceae* and fecal LL-37 levels was observed. Similarly, *Lachnospira* and *Ruminococcus* (Lachnospiraceae) were found to correlate with β-defensins 2 levels. Furthermore, FM-CBAL74 supplementation resulted in an increase in the relative abundance of genes involved in butyrate synthesis, especially genes encoding butyryl-CoA transferase (EC 2.8.3.8) and butyrate kinase (EC 2.7.2.7). Consistently, a significant increase in fecal butyrate levels in children receiving FM-CBAL74 dietary supplementation, likely deriving from lactate catabolism one of the primary pathways for butyrate production by gut bacteria [19].

Analyzing the raw data of the three clinical studies, Calame et al. demonstrated that the baseline values were able to influence the α-defensin, β-defensin 2, LL-37, sIgA, and butyrate increase after three months of FM-CBAL74 dietary supplementation [20].

To evaluate the association between fecal immune biomarkers and the number of AGE, we performed Spearman’s correlation on raw data from the two clinical trials [14,15]. A significant negative association between the increase in α-defensin (rho − 0.271, *p* = 0.002), β-defensin (rho − 0.217, *p* = 0.012), and LL-37 (rho − 0.177, *p* = 0.038), and the number of AGE episodes was observed. In addition, a significant negative association between the increase in sIgA and the total number of AGE was observed (rho − 0.258, *p* = 0.001). Similarly, a significant negative association between the change in fecal butyrate and the total number of AGE was observed (rho − 0.493, *p* = 0.027).

### 5.4. Limitations of the Available Clinical Data

The main limitations of all available studies derive from the lack of identification of which FM-CBAL74 components could be responsible for the protective effects, of clinical data on the dose-dependency of the reported beneficial effects, of data on long-term tolerability, and of clinical efficacy in subjects with different ages.

## 6. Toward a Research Agenda

In both RCTs performed in children aged 1–4 years a protective action elicited by FM-CBAL74 against acute respiratory tract infections was also demonstrated [14,15]. These findings open the way to future research activities aimed at the definition of the beneficial action of dietary supplementation with FM-CBAL74 against extra-intestinal infections. More research is also needed to better define the optimal dose and timing for the dietary supplementation with this postbiotic. Additional investigations are advocated to better define the specific components of FM-CBAL74, which could be responsible for the observed protective actions. Lastly, future research would be necessary to better define the mechanisms of these protective effects using more complex systems, such as human biopsies and/or organoids exposed to different gastrointestinal pathogens.

## 7. Conclusions

Emerging preclinical and clinical evidence support the potential role of dietary supplementation with FM-CBAL74 for the prevention of pediatric intestinal infections. Available data are summarized in Table 1.

**Figure 7:**
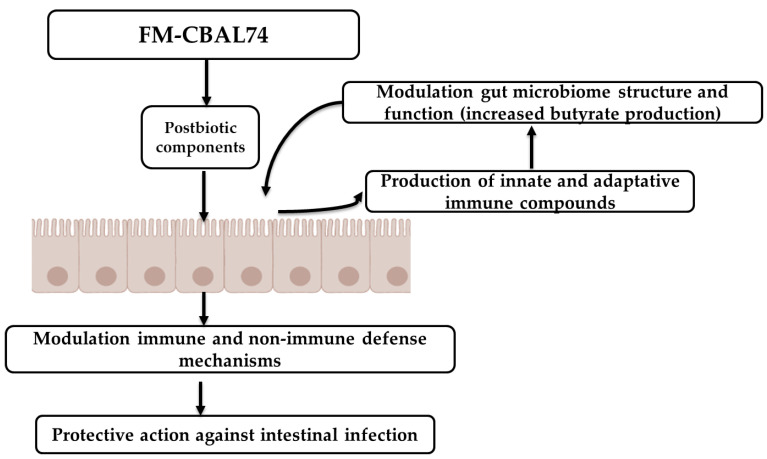
**FM-CBAL74 modulates different defense mechanisms at intestinal level.** The available literature suggests the potential role of dietary supplementation with FM-CBAL74 for the prevention of pediatric intestinal infections. The effects could be related to the activity of several components, including bacterial DNA and cell membrane components (e.g., peptidoglycans, polysaccharides, and lipoteichoic acid), as well as bacterial produced products during the fermentation, such as peptides and metabolites (e.g., lactic acid and other organic acids).

This dietary intervention seems to be safe and well-accepted by neonates, infants, and children. The available literature suggests that these beneficial clinical effects could derive from a modulation of several defense mechanisms at the intestinal level. The effects could be related to the activity of several components, including bacterial DNA and cell membrane components, as well as bacterial products produced during the fermentation process, such as peptides and metabolites (e.g., lactic acid and other organic acids). Altogether, these findings suggest that FM-CBAL74 is an innovative preventive and therapeutic strategy against pediatric gastrointestinal infectious diseases (Figure 7).

## Figures and Tables

**Table 1:** A summary of the most relevant preclinical and clinical evidence on the protective action elicited by the postbiotic FM-CBAL74 against pediatric intestinal infections.

Preclinical Evidence		
Outcomes	Effects	References
Modulation of the major defense mechanisms against intestinal infections	Stimulation of enterocytes cell growth and differentiation, tight-junctions proteins expression, mucus layer thickness, and innate immunity peptides production.	[11]
Anti-inflammatory activity	Inhibition of pro-inflammatory cytokines release, and modulation of Toll-like receptor 2 and 4, transcriptional factor nuclear factor kappa B1, and trefoil factor 3 expression in enterocytes and dendritic cells.	[11,12,17]
Anti-viral action	Protection against *Rotavirus*-induced gut barrier damage, oxidative stress, and pro-inflammatory cytokines release. Protection against SARS-CoV-2 entry into human enterocytes, and pro-inflammatory cytokines release	[12,13]
Clinical evidence		
Protection against pediatric acute gastroenteritis	Significant reduction of the rate of children presenting ≥ 1 episodes of acute gastroenteritis and of the total number of acute gastroenteritis episodes.	[14,15]
Stimulation of innate and adaptative immunity	Increased intestinal production of α- and β-defensins, cathelicidin LL-37, and secretory immunoglobulin A.	[14,15,16]
Modulation of gut microbiome	Beneficial modulation of gut microbiome structure and function in formula fed neonates, infants, and children with increased abundance of healthy butyrate-producer bacteria.	[16,19]

## Data Availability

The datasets analyzed are available from Roberto Berni Canani upon request (berni@unina.it).
